# Taxifolin Inhibits WSSV Infection and Transmission by Increasing the Innate Immune Response in *Litopenaeus vannamei*

**DOI:** 10.3390/v14122731

**Published:** 2022-12-07

**Authors:** Xu Zhang, Li-Peng Shan, Qi Zhao, Lei Liu, Xu OuYang, Yang Hu, Chen-Jie Fei, Jiong Chen

**Affiliations:** 1 State Key Laboratory for Managing Biotic and Chemical Threats to the Quality and Safety of Agro-Products, Ningbo University, Ningbo 315832, China; 2 Laboratory of Biochemistry and Molecular Biology, School of Marine Sciences, Meishan Campus, Ningbo University, Ningbo 315832, China; 3 Key Laboratory of Applied Marine Biotechnology of Ministry of Education, Meishan Campus, Ningbo University, Ningbo 315832, China; 4 Zhejiang Zhengda Livestock, Poultry and Aquatic Products Co., Ltd., Ningbo 315806, China

**Keywords:** taxifolin, WSSV, antiviral, Chinese herbs, *Litopenaeus vannamei*

## Abstract

An outbreak of white spot syndrome virus (WSSV) can hit shrimp culture with a devastating blow, and there are no suitable measures to prevent infection with the virus. In this study, the activity of active molecules from Chinese herbs against WSSV was evaluated and screened. Taxifolin had the highest rate (84%) of inhibition of the WSSV infection. The viral infectivity and genome copy number were reduced by 41% when WSSV virion was pretreated with taxifolin prior to shrimp infection. A continuous exchange of taxifolin significantly reduced the mortality of shrimp infected with WSSV. Due to the WSSV virion infectivity being affected by taxifolin, the horizontal transmission of the virus was blocked with an inhibition rate of up to 30%, which would further reduce the cost of a viral outbreak. Additionally, the viral genome copy number was also reduced by up to 63% in shrimp preincubated in taxifolin for 8 h. There may be a connection to the enhancement of innate immunity in shrimp that resulted in a 15% reduction in mortality for taxifolin-fed shrimp after the WSSV challenge. After dietary supplementation with taxifolin, the resistance of larvae to WSSV was improved, indicating that taxifolin may be a potential immunostimulant for shrimp to prevent WSD. Therefore, the results indicate that taxifolin has application potential for blocking a WSSV outbreak and reducing the loss of shrimp culture.

## 1. Introduction

To meet the demand for protein of a growing population, the aquaculture industry has developed rapidly and is deemed to be the sector with the most potential for protein product supply [1]. Within the aquaculture industry, the crustacean industry is growing at an average of 16% a year, accounting for more than 30% of the total profit from aquaculture, producing over USD 76.3 billion (10.4 million tonnes) in 2019 [2]. Nevertheless, it has experienced outbreaks of various viral diseases, which have brought challenges to the rapid and stable progress of crustacean culture production. White spot syndrome virus (WSSV) is highly challenging because there are still no suitable measures for its prevention or control [3,4]. Due to the variety of hosts, complex transmission routes and high mortality and morbidity, the virus causes white spot disease (WSD), which is so severe around the world that it is reportable to OIE [5,6,7,8]. Therefore, there is an urgent and important need to develop an efficient strategy for a primary intervention for WSSV infection.

As a traditional control method, synthetic chemical drugs such as antibiotics are used to improve the tolerance of shrimp to WSD [9,10]. Although the desired effect is achieved in a short time, the negative effects, such as drug residues and environmental pollution, pose a threat to human health [11,12,13]. Therefore, it is critical to find an efficient, safe and non-polluting control method to increase the resistance of shrimp to WSSV. This social need has led researchers to focus on alternatives with fewer adverse reactions, fewer residues and less environmental pollution and resistance, which has accelerated the search for beneficial plants for this purpose. There is a long history of using Chinese herbs to block disease outbreaks in aquaculture because of the numerous existing active small molecules in plants [14,15]. Previous research mainly concentrated on blocking a WSSV outbreak with the use of crude extracts of medicinal plants. For example, *Argemone Mexicana* can reduce the damage of WSSV by enhancing the innate immune response of shrimp [16]. The mortality rate of WSSV-infected *Penaeus monodon* was reduced after feeding crude extracts of Chinese herbs [17]. However, complex active ingredients in anti-WSSV extracts generally show a low economic benefit and adverse factors, which makes it difficult to apply in aquaculture [18]. Consequently, many researchers prefer to screen the effective antiviral molecules of the herbs because of their clearer structure and higher stability [19,20,21,22].

The innate immune system of shrimp is essential to defend against foreign pathogens. However, shrimp larvae are in an early stage of development, and their innate immune system is not fully mature, which makes them susceptible to WSSV infection [23]. Since vaccination in this process is negligible, it is important to develop an immunostimulant for controlling WSD outbreaks [8]. Some earlier studies have verified that active small molecules from Chinese herbs improve the survival time of shrimp infected with WSSV by activating the non-specific immune response [17,24,25]. Here, a total of ten active small molecules were evaluated for their anti-WSSV activities in *L. vannamei* larvae. Among these molecules, taxifolin was determined to have the highest anti-WSSV activity, as well as demonstrating an increase in WSSV-infected shrimp survival. The data suggested that taxifolin suppressed the horizontal transmission of WSSV and induced the innate immune response of the larvae, which is useful for blocking the outbreak of WSSV in aquaculture.

## 2. Materials and Methods

### 2.1. Shrimp, WSSV and Medicines

*Litopenaeus vannamei* (body length: 5.5 ± 0.2 mm, body weight: 4.7 ± 0.6 mg) and WSSV-infected shrimp were donated by the Zhejiang Mariculture Research Institute (Wenzhou, China) and temporarily kept in culture ponds and fed commercial feed four times a day. Prior to the experiment, the larvae were collected to verify that they were not infected with WSSV by PCR testing. The conditions were 92 °C 4 min; 92 °C 20 s, 62 °C 20 s, and 72 °C 30 s for 35 cycles; 72 °C 3 min. The products were analyzed with 1.5% (*w*/*v*) agarose gel electrophoresis [26]. In order to ensure the efficiency and authenticity of infection, the shrimp infected with WSSV were ground and fed to healthy shrimp to infect with WSSV. Briefly, the WSSV-infected shrimp were ground to extract DNA; afterward, the viral copy levels were analyzed by qPCR. Aquacultural water was used to dilute the homogenates to a copy level of 1.6 × 10^5^ copies/μL, followed by infecting the healthy shrimp [26]. The screening criteria for active small molecules were those that had been reported to have antiviral and immune-enhancing activities but with unknown anti-WSSV activity. A total of ten active small molecules were purchased from Aladdin (Shanghai, China) to dissolve in 100% DMSO (Beyotime, Shanghai, China) as the stock solution at 100 mM. The active small molecule stock solution described above was processed by ultrasonic dissolution for 10 min to ensure full dissolution.

### 2.2. Acute Toxicity of Molecules on Shrimp

The shrimp were placed in 6-well plates with 10 shrimp per well and subsequently exposed to the active small molecules that had been continuously diluted to obtain final concentrations of 1, 10, 20, 30, 40, 50, 60, 70, 80, 90 and 100 μM, respectively. DMSO (1‰, V_DMSO_/V_Water_) was added to the aquacultural water as a control. The status of the larvae was observed every 24 h, and the dead shrimp were taken out in time to avoid affecting the water quality.

### 2.3. Anti-WSSV Effect of Medicines

In order to screen the best antiviral activity from the ten active small molecules, co-incubation was conducted on 6-well plates with 10 shrimp larvae in each well. Molecules (safe concentration) and virus (1.6 × 10^5^ copies/μL) were mixed into the well with the shrimp and incubated for 72 h. Shrimp were co-incubated with DMSO (1‰, V_DMSO_/V_Water_) and WSSV as control. Finally, the larvae were collected, and the virus copy levels were analyzed according to reported methods [22]. Briefly, a fragment of *VP28* in the WSSV genome was amplified and inserted into the plasmid, then qPCR was performed after gradient dilution. PCR procedure: 95 °C 5 min, 95 °C 10 s for 40 cycles, 55.5 °C 30 s. The copy number was estimated by the molecular weight of the plasmid [27]. Finally, standard curves of cycle thresholds and WSSV genome copy numbers were plotted for subsequent experiments. TIANamp Marine Animals DNA Kit (Tiangen Biochemical Technology Co., Ltd., Beijing, China) and Nucleic acid analyzer (Nano-200, Hangzhou Aosheng Instrument Co., Ltd., Hangzhou, China) were used to extract DNA and detect the concentration, and then the copy levels of WSSV was calculated by qPCR.

To verify the potential positive effect of taxifolin on virions, the WSSV (1.6 × 10^5^ copies/μL) was pretreated with taxifolin (100 μM) in challenge wells. After being preincubated for 1, 3 and 6 h, the shrimp were randomly placed into challenge wells and infected for 72 h. After that, the shrimp were euthanized using tricaine methanesulfonate (MS-222, 150 mg/L); afterward, the virus genome copy number was quantified.

For preincubation, the potential preventive effect of taxifolin on WSSV resistance of shrimp larvae was explored. The larvae were preincubated with taxifolin (100 μM) for 2, 4, 6 and 8 h, then challenged with virus for 72 h and simultaneously removed from the taxifolin. The surviving shrimp were euthanized by MS-222, and the WSSV copy levels were calculated as above.

The effects of drug metabolism and environmental degradation on the antiviral activity of taxifolin were analyzed by continuous immersion. Shrimp were infected with virus in challenge wells for 24 h, then water containing the WSSV in each well was changed to a solution of taxifolin (100 μM) or DMSO (1‰, V_DMSO_/V_Water_) for further incubation. The solution in the wells was replaced with a new solution of taxifolin or DMSO every 24 h in subsequent experiments. The surviving shrimp were collected every 24 h and euthanized with MS-222 and immediately stored in liquid nitrogen until the WSSV copy levels were detected by qPCR.

### 2.4. Stability of Taxifolin

To explore the effective retention time of taxifolin in aquacultural water and find the appropriate treatment cycle, a stability assay was conducted. The water was obtained from the shrimp larvae cultivation pool. Taxifolin (100 μM) was mixed into the water and placed in a natural environment for 1~3 d. Daily, the aqueous solution of taxifolin was premixed with virus and coinfected shrimp and stored for 72 h. The survival status of larvae was observed each 24 h, and the virus copy level was measured at 72 h.

### 2.5. Effect of Taxifolin on Horizontal Transmission of WSSV

The shrimp larvae without WSSV infection were incubated in the WSSV environment for 24 h to acquire the diseased shrimp (donor), which were subsequently transferred to a beaker containing taxifolin (100 μM) solution. At the same time, healthy shrimp (recipient) were placed in the same beaker and separated by a net so that there was no direct contact between the donor and recipient. The diseased shrimp (donor) and healthy shrimp (recipient) were placed in the same beakers containing DMSO (1‰, V_DMSO_/V_Water_) solution and separated by net as control. After 72 h of co-incubation, the recipient shrimp were collected to detect the copy number of the WSSV genome.

### 2.6. Effect of Taxifolin on Innate Immunity of Shrimp

To investigate the relationship between taxifolin and the immune response of shrimp, larvae without WSSV infection were exposed to taxifolin (at a final concentration of 100 μM) for 120 h. DMSO (1‰, V_DMSO_/V_Water_) was served. Three replicates were set up in this experiment. After that, shrimp larvae were collected, and the relative expression levels of immune-related genes were detected in accordance with previous methods [28,29]. Briefly, Trizol reagent (Takara, Kusatsu, Japan) and NanoDrop spectrophotometers (ThermoFisher, Waltham, MA, USA) were used to extract total RNA and to examine the quality. The RNA purity was evaluated by the absorbance ratio of A260 nm/A280 nm, which ranged from 1.8 to 2.0. A total of 500 ng/μL of RNA was used for cDNA generation per reaction, which was synthesized by HiScript Q Select RT SuperMix (Vazyme, Nanjing, China). All primers in this experiment were provided by Shanghai Sangong Bioengineering Co., Ltd. (Shanghai, China), and the primer information is detailed in Table 1. The reference gene was *EF1α*, and the ChamQ^TM^ SYBR^®^ qPCR Green Master Mix (Vazyme, Nanjing, China) was used on an ABI StepOnePlus^TM^ Real-Time PCR system (ThermoFisher, Waltham, MA, USA). The PCR procedure was 95 °C 30 s, 95 °C 10 s 40 cycles, 60 °C 30 s. To evaluate the specificity of each amplicon, melt curve analysis was performed at the end of each thermal profile. No template control (NTC) and no-RT control were set during PCR. The final gene expression was analyzed by 2^−ΔΔCT^ [30].

### 2.7. Effects of Dietary Supplementation with Taxifolin on WSSV Replication

Taxifolin was dissolved in sterilized aquacultural water to obtain a solution (10 mg/mL), and afterward, it was sonicated for 10 min to ensure the taxifolin was fully dissolved and mixed. The solution was evenly sprayed into commercial feed (Yongdeli Feed Co., Ltd., Chaozhou, China: crude protein ≥ 43%, crude lipids ≥ 6%, ash ≤ 16%, crude fiber ≤ 3%) at a concentration of 1 g/kg, followed by drying, sieved through an 80-mesh screen and sealing for storage at 4 °C [34,35]. Healthy shrimp larvae were cultured in a beaker (containing 30 larvae and 500 mL aquacultural water) and fed with commercial feed containing taxifolin every 6 h. There was timely removal of dead shrimp and daily replacement of aquaculture water to prevent water deterioration. After 7 days of feeding, the larvae described above were placed with WSSV for 72 h. During the 72 h WSSV infection, the shrimp in the experimental group continued to be fed the commercial feed containing taxifolin, while the control group was fed the commercial feed without taxifolin. The survival status of the larvae was recorded every 24 h, and the larvae were randomly collected to detect the virus genome copy levels.

### 2.8. Statistical Analysis

The GraphPad Prism 7.0 (Graphpad Software, Inc., San Diego, CA, USA) and SPSS 18.0 (SPSS, Chicago, IL, USA) software were used for image rendering, data statistics and significance analysis. All the values are shown as the mean ± SD, and *p* < 0.05 was considered a significant difference.

## 3. Results

### 3.1. Preliminary Screening on Active Small Molecules

**Figure 1:**
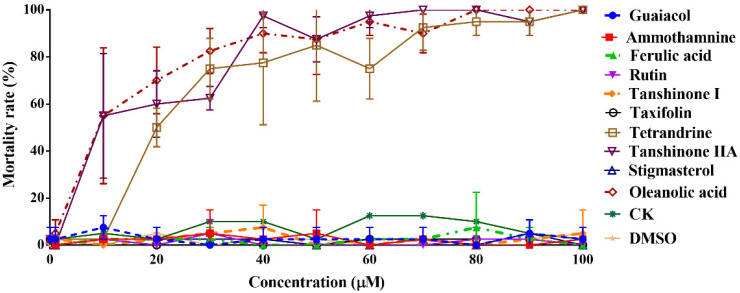
Medicinal toxicity was analyzed in vivo. Shrimp larvae were incubated with ten active small molecules of 0–100 μM for 72 h, and the mortality rate was analyzed. Each value was represented as the mean ± SD.

**Figure 2:**
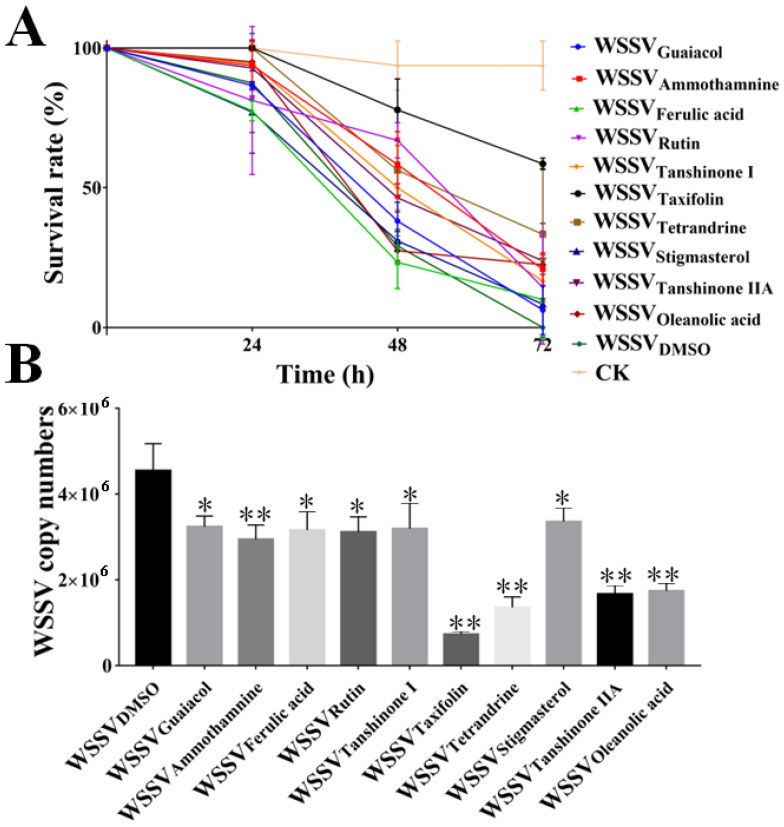
Anti-WSSV activity of taxifolin was the best. (**A**) Survivorship curves of shrimp larvae were co-incubation with active small molecules (safe concentration) and WSSV for 72 h. (**B**) The copy number of WSSV genome was detected by qPCR at 72 h. Each value was represented as the mean ± SD. The *p* value was determined by Student’s *t*-tests. ** *p* < 0.01; * *p* < 0.05.

We screened the safe concentration of the molecules and determined that it was non-toxic to the larvae. The mortality rate of larvae incubated with ten active small molecules for 72 h is shown in Figure 1. Safe concentrations of molecules were analyzed based on the mortality rate of the larvae. The results showed that the safe concentrations of molecules as follows: guaiacol (100 μM), ammothamnine (100 μM), ferulic acid (100 μM), rutin (100 μM), tanshinone I (10 μM), taxifolin (100 μM), tetrandrine (10 μM), stigmasterol (100 μM), tanshinone IIA (1 μM) and oleanolic acid (1 μM). Next, we evaluated whether molecules could inhibit WSSV at previously established safe doses. Antiviral data showed that taxifolin had the highest anti-WSSV effect, reducing the proportion of WSSV-infected shrimp dying by 41% and decreasing WSSV loads up to 84%. In contrast, tetrandrine, tanshinone IIA and oleanolic acid also inhibited the replication of the virus in WSSV-infected larvae by 70%, 63% and 62%, respectively (Figure 2). Therefore, taxifolin was selected for further study of its promising activity.

### 3.2. Anti-WSSV Activity of Taxifolin

**Figure 3:**
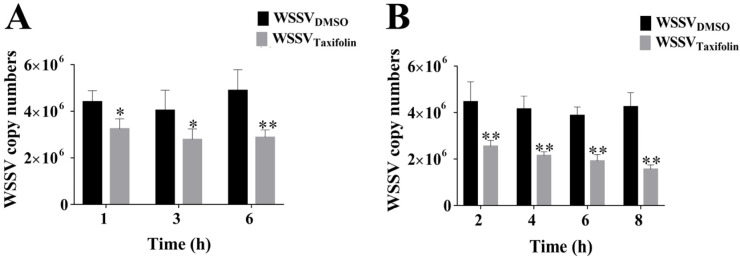
The replication of WSSV was inhibited by preincubation of WSSV virions and shrimp with taxifolin. (**A**) Taxifolin (100 μM) was preincubated with the WSSV virions for 1, 3 and 6 h, respectively. Inhibition was enhanced within an increased exposure time of taxifolin to the virus. (**B**) Concentrations of up to 100 μM taxifolin were pre-exposed to shrimp larvae for 2, 4, 6 and 8 h, respectively, followed by WSSV infection. Each value was represented as the mean ± SD normalized to values for no treatment. The *p* value was determined by Student’s *t*-tests. ** *p* < 0.01; * *p* < 0.05.

Meanwhile, to determine whether virions could be destroyed by taxifolin and thus reduce WSSV infection, we carried out further preincubation on the WSSV virions with taxifolin. Before infecting the larvae, WSSV was pretreated with taxifolin for 1, 3, and 6 h. The effect of taxifolin on inhibiting WSSV infection increased with the prolongation of preincubation time (Figure 3A). There was significant inhibition of virus replication (WSSV at 1.6 × 10^5^ copies/μL) with 1 h and 3 h of preincubation time, and the highest inhibition was with 6 h preincubation time (*p* ≤ 0.01), with an inhibition rate of 41%. Taxifolin was directly applied to shrimp larvae, and then they were infected with untreated WSSV. The inhibition of taxifolin on WSSV replication was also positively correlated with preincubation time (Figure 3B). Compared with WSSV/taxifolin preincubation, the inhibition of WSSV replication was slightly increased in larvae/taxifolin preincubation, and the inhibition rate of taxifolin on virus copy was the highest at 8 h (63%). WSSV replication was inhibited in all treatment groups. Due to the promising results, especially in the larvae preincubated with taxifolin, we conducted subsequent experiments on the stability of taxifolin.

### 3.3. The Stability of Taxifolin

**Figure 4:**
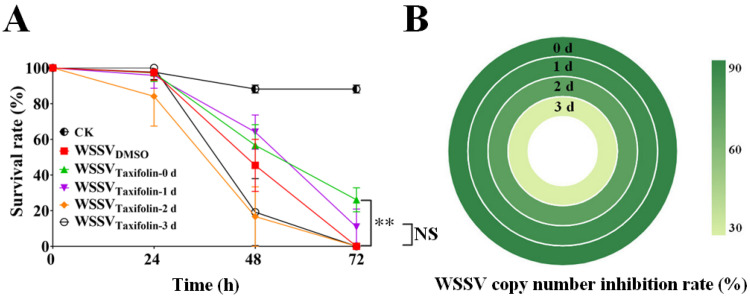
The half-life of taxifolin in aquacultural water was short. Taxifolin (100 μM) was added to water and placed at 28 °C for 0–3 d. On the final day, WSSV was premixed with each water sample to infect shrimp larvae. (**A**) Survivorship curves of WSSV-infected shrimp. (**B**) Quantification of copy number of the WSSV genome at 72 h was measured by qPCR. Each value was represented as the mean ± SD. The *p* value was determined by Student’s *t*-tests. ** *p* < 0.01; NS *p* > 0.05.

In this experiment, the stability of taxifolin in water was tested. Taxifolin was added to water samples (in order to simulate the environment of real aquaculture, water samples were taken from the shrimp larvae cultivation pool). The samples were placed for approximately one to three days at 28 °C (with exposure to natural light) and premixed with WSSV, followed by co-incubated shrimp larvae. Taxifolin was relatively stable in water at 28 °C and was less efficacious with the increase of time in water samples. Data showed that taxifolin was significantly inactivated from day two to three; that is, shrimp larvae all died (Figure 4A), or the WSSV replication inhibition rate decreased significantly (Figure 4B). These results indicated that the half-life of taxifolin in aquacultural water was short, and continuous immersion of taxifolin seems to be an effective method for reducing WSSV infection.

### 3.4. Effect of Continuous Taxifolin Treatment on WSSV Replication

**Figure 5:**
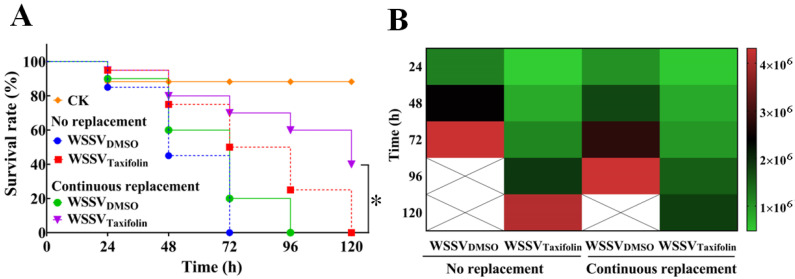
Continuous treatment of taxifolin further inhibited WSSV replication in vivo. Aquacultural water and new taxifolin were exchanged or non-exchanged every 24 h, respectively. (**A**) Survivorship curves of WSSV-infected shrimp were analyzed. (**B**) Quantification of copy number of the WSSV genome was tested every 24 h. The *p* value was determined by Student’s *t*-tests. * *p* < 0.05.

In order to verify our conjecture above, WSSV-infected shrimp were treated continuously with taxifolin, and an expected result showed that more than 50% of the shrimp were still alive after 72 h of taxifolin treatment, while all the shrimp without taxifolin treatment died at 72 h (Figure 5A). Surprisingly, 40% of the WSSV-infected shrimp were still alive after 120 h of continuous treatment with taxifolin, at which time the viral copy inhibition rate was 57% (Figure 5B). To our surprise, a continuous exchange of water also seemed to alleviate WSSV damage to larvae. In this study, all WSSV-infected shrimp died after 96 h of continuous exchange of water (Figure 5A). Undoubtedly, these results confirm our suspicion that continuous treatment was an important method for enhancing the anti-WSSV activity of taxifolin.

### 3.5. Taxifolin Inhibits Horizontal Transmission of WSSV

**Figure 6:**
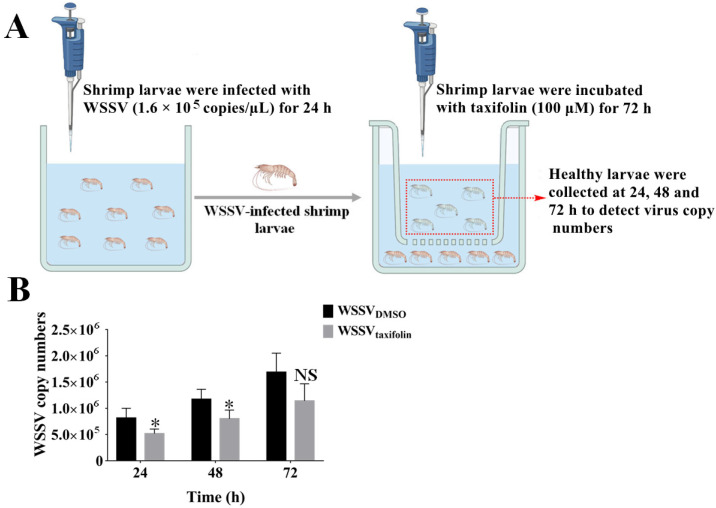
Taxifolin blocked the horizontal transmission of WSSV. (**A**) Schematic diagram describing the workflow. (**B**) WSSV genome copy number of shrimp larvae was tested every 24 h. Each value was represented as the mean ± SD. The *p* value was determined by Student’s *t*-tests. NS *p* > 0.05; * *p* < 0.05.

The main reason for a large-scale outbreak of WSSV and the difficulty of effective prevention and control is horizontal transmission among shrimp [36,37]. Based on these results, we explored the ability of taxifolin to inhibit horizontal transmission of WSSV. The diseased shrimp (donor) were placed in a beaker containing a solution of taxifolin (100 μM), while the healthy shrimp (recipient) were placed in the same beaker and separated with a net to ensure no direct contact (Figure 6A). Here, the results revealed that the virus copy number in the recipient shrimp treated with taxifolin was lower than in the recipient shrimp without taxifolin treatment at different times (Figure 6B). Among them, taxifolin showed the strongest inhibitory effect on WSSV copy at 24 h, with an inhibition rate of 36%. In this study, the horizontal transmission of WSSV between shrimp was effectively inhibited by taxifolin but not completely blocked.

### 3.6. Effects of Taxifolin on Expression of Immune-Related Genes

**Figure 7:**
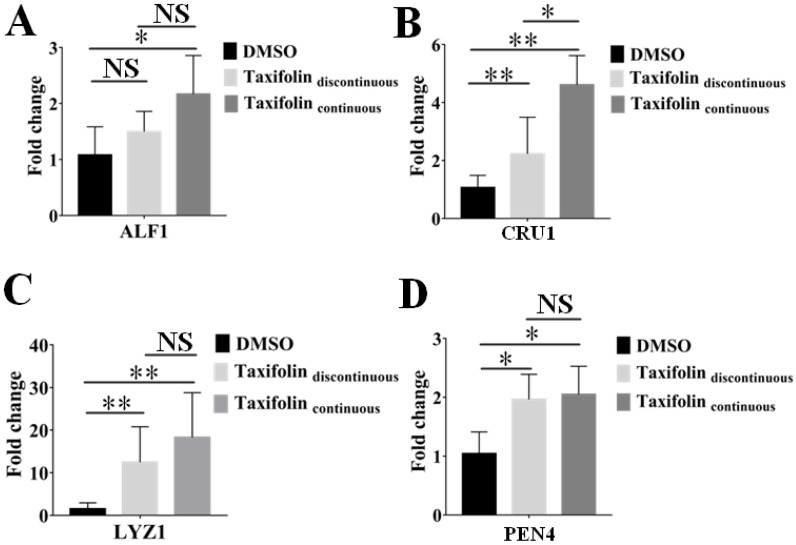
The expression level of immune-related genes in shrimp was increased by taxifolin. The innate immune response of shrimp larvae was analyzed after exchanged or non-exchanged new taxifolin for five days. The relative RNA levels of *ALF1* (**A**), *CRU1* (**B**), *LYZ1* (**C**), and *PEN4* (**D**) were determined by qPCR at 120 h. Each sample was run in triplicate normalized to shrimp *EF1α*. Each value was represented as the mean ± SD normalized to values for no treatment. The *p* value for each study was determined by Student’s *t*-tests. ** *p* < 0.01; * *p* < 0.05; NS *p* > 0.05.

To determine whether taxifolin induces an immune response in shrimp and thus has potential use as an immunostimulant, we measured immune-related gene expression levels in shrimp exposed to taxifolin. As expected, the expression of immune-related genes was upregulated in shrimp incubated with taxifolin for 120 h in comparison with the control group. Similarly, this promotion was further enhanced after the continuous change of taxifolin treatment for 120 h (Figure 7). The expressions of *LYZ1*, *PEN4* and *CRU1* in larvae immersed in taxifolin (100 μM) for 72 h were significantly increased. After continuous replacement of taxifolin treatment for 120 h, the relative expressions of *ALF1*, *CRU1*, *LYZ1*, and *PEN4* were elevated 1.0-, 3.4-, 7.3-, and 1.0-fold over the control group, respectively. Our results suggest that taxifolin may protect the larvae by activating the non-specific immune system.

### 3.7. Dietary Supplementation with Taxifolin Inhibited WSSV Replication in Shrimp

**Figure 8:**
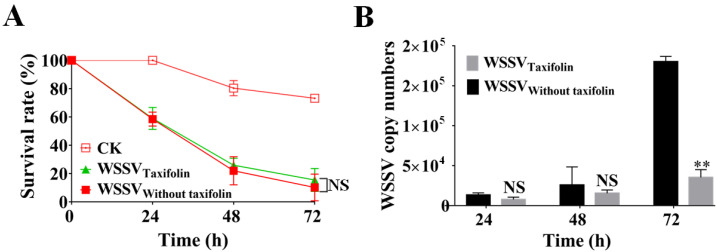
Dietary supplementation with taxifolin could inhibit the WSSV replication in shrimp larvae. After seven days of dietary supplementation with taxifolin, shrimp larvae were challenged with WSSV. Afterward, the WSSV-infected shrimp continued to be fed with or without taxifolin for three days. (**A**) The survival rate of WSSV-infected shrimp was analyzed. (**B**) Quantification on copy number of WSSV genome was tested every 24 h. Each value was represented as the mean ± SD. The *p* value was determined by Student’s *t*-tests. ** *p* < 0.01; NS *p* > 0.05.

In order to evaluate whether dietary supplementation of taxifolin can enhance the WSSV resistance of shrimp, we added taxifolin into the commercial feed for feeding shrimp larvae. As expected, dietary supplementation with taxifolin improved the resistance of larvae to WSSV and resulted in an increased survival rate of taxifolin-feeding shrimp by more than 10% after a 72 h WSSV challenge. Interestingly, WSSV-infected shrimp fed with commercial feed containing taxifolin further improved their survival rate; nevertheless, the promoting effect was not significant (Figure 8A). The results also showed that dietary supplementation of taxifolin during WSSV infection effectively blocked replication of the viral genome in WSSV-infected shrimp. The virus copy number was significantly increased at 72 h in larvae that were not fed with taxifolin during WSSV infection (Figure 8B). This suggested that dietary supplementation of taxifolin had the potential to prevent WSSV outbreaks in aquaculture.

## 4. Discussion

Remarkably, active small molecules isolated from Chinese herbal medicine can be studied in depth because of their clear structure, and are natural and safe, which is considered important for developing new environmentally friendly WSSV control agents [20,22]. Previous data have demonstrated that esculin, geniposidic acid, coumarin derivatives and other active small molecules isolated from Chinese herbal medicine have encouraging activity in preventing WSSV outbreaks [8,22,38]. However, the complex structure, poisonous effects and instability in aquacultural water limit their practical application in aquaculture. Therefore, it is urgent to screen active small molecules isolated from Chinese herbal medicines with simpler structures, lower toxicity and more stable activity for application in shrimp culture. In this study, ten active small molecules from Chinese herbal medicines were screened, and taxifolin was determined to have the best anti-WSSV activity, which could reduce the proportion of WSSV-infected shrimp dying by 41% and decrease WSSV loads up to 84%. Different experimental methods were used to further evaluate the anti-WSSV activity of taxifolin.

The main methods to block virus replication include destroying virus virions, blocking virus infection processes and inhibiting virus assembly in the host [39]. In this research, the preincubation of taxifolin in shrimp did not completely prevent WSSV infection but still inhibited the replication of WSSV in shrimp. It may be that the invasion and transmission of WSSV were influenced by the action of taxifolin on specific targets [40,41]. Similarly, based on reported studies, we speculated that taxifolin might have an effect on WSSV virions [19,39,40,41,42]. Excitingly, the WSSV virions were pretreated with taxifolin before infecting shrimp, and it was found that the replication of WSSV in shrimp was reduced, while prolonged preincubation time enhanced this inhibitory effect. The results confirmed our conjecture. In summary, it is reasonable to believe that taxifolin could exert its unique anti-WSSV activity by blocking the invasion and transmission of WSSV and destroying the virus virions.

The aquacultural environment is affected by temperature, light, algae, microorganism, residual bait and many other factors that determine the degradation and activity of antivirals [26]. It was found that the antiviral effect of taxifolin was weakened gradually when it was degraded in water. Therefore, long-term continuous treatment has become an important method to block the deterioration of WSSV [26,43]. In this experiment, treatment of WSSV-infected shrimp with taxifolin resulted in prolonged survival time and blocked the replication of WSSV. Surprisingly, continuous treatment with taxifolin extended the survival time of WSSV-infected shrimp even further. This phenomenon implies that taxifolin has a protective effect on WSSV-infected shrimp, and the continuous use of taxifolin is the key to inhibiting an outbreak of WSSV. More meaningful, a continuous exchange of water was shown to attenuate WSSV damage to larvae and increase the survival time of shrimp infected with WSSV, which could be an effective method to alleviate the economic loss caused by WSSV in shrimp cultures. There are two modes of WSSV transmission, horizontal transmission and vertical transmission. Among them, horizontal transmission refers to the transmission through ingestion of diseased shrimp tissues or contact with aquacultural water containing WSSV. Studies have shown that this mode of transmission has been the primary cause of WSSV outbreaks [36,37]. In this research, taxifolin effectively blocked the horizontal transmission of the virus between shrimp, suggesting the potential of the application of taxifolin in blocking WSSV outbreaks.

It was reported that active small molecules from plants or their metabolites acting as immunostimulants could activate the innate immune system and reduce the replication of pathogens [44]. For example, adding a compound of Chinese herbal medicine to the diet of shrimp for 30 days could enhance their resistance to *Vibrio parahemolyticus* by improving the expression of immune-related genes [45]. Furthermore, shrimp-fed extracts of Chinese herbs such as *Rheum palmatum* L. and *Andrographis paniculata* improved immune-related enzyme activities such as ACP, ALP, SOD, UL and UA [46]. Yu-ping-feng polysaccharides, as an immunostimulant, promoted the growth and improved the immunity of *Ctenopharyngodon idella* [47]. Similar outcomes were found for *Gardenia Jasminoides*, *Argemone Mexicana*, *Cyanodon Dactylon* and *Aegle Marmelos* [16,17,48]. The adaptive immune system of shrimp is not mature, so the innate immune system is essential for defending against invading pathogens [49]. When pathogens invade shrimp, they are recognized by PRRs and activate humoral and cellular immunity [50]. The innate immune response of WSSV is mainly mediated by antimicrobial peptides (AMPs) synthesized by secreting cells and secreted into body fluid. Data has shown that AMPs are closely related to the antibacterial and antiviral processes in shrimp [51,52,53,54]. Here, we incubated shrimp with taxifolin and detected the relative expression of immune-related genes. As expected, taxifolin promoted the expression of *ALF1*, *CRU1*, *LYZ1* and *PEN4* in shrimp, suggesting that taxifolin exerts its anti-WSSV activity by activating the immune system of shrimp.

After evaluation in multiple experiments, we have again demonstrated the anti-WSSV activity of taxifolin. In the future, we will investigate the practical application of taxifolin in shrimp culture by studying the administration methods. The main methods of administration in aquaculture include injection, smear, oral and immersion bath, among which injection, smear and immersion bath are not suitable for large-scale operations; therefore, oral is the most feasible method of administration [55,56]. In this research, the shrimp larvae were fed with taxifolin for seven days, followed by infection with WSSV. The data shows that dietary supplementation of taxifolin increases the resistance of shrimp to WSSV. However, continuing to feed taxifolin to WSSV-infected shrimp did not significantly improve their survival rate, which may be caused by reduced or ceased intake of shrimp after infection with WSSV [57,58]. The above data indicate that it is possible to add taxifolin to feed for the prevention of a WSSV outbreak.

## 5. Conclusions

**Figure 9:**
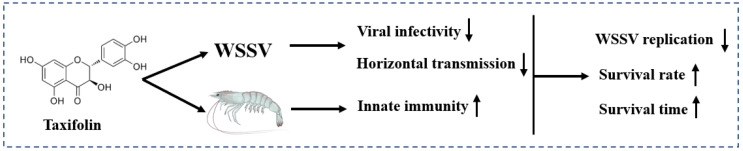
Schematic illustration of anti-WSSV activity of taxifolin in shrimp. **↑** up-regulation; **↓** down-regulation.

In conclusion, the anti-WSSV activity of taxifolin was the best among the 10 active small molecules, which could effectively prolong the survival time of shrimp infected with WSSV and showed significant anti-WSSV activity. Continuous treatment of taxifolin further reduced the replication level of the virus and reduced the proportion of dead shrimp infected with WSSV. Taxifolin also blocked the horizontal transmission of the virus and improved the non-specific immunity of the shrimp. Adding taxifolin to the diet could enhance the WSSV resistance of shrimp, supporting the feasibility of using taxifolin to prevent a WSSV outbreak (Figure 9). Therefore, taxifolin, as an immunostimulant, has a broad prospect in blocking outbreaks of WSSV.

## Figures and Tables

**Table 1:** Sequences of primer pairs used for analysis of gene expression by qRT-PCR.

Genes		Primer Sequence (from 5′ to 3′)	Amplicon (bp)	Accession No. [31,32,33]
EF1α	Forward	GTATTGGAACAGTGCCCGTG	143	GU136229
	Reverse	ACCAGGGACAGCCTCAGTAAG		
ALF1	Forward	TTACTTCAATGGCAGGATGTGG	142	EW713395
	Reverse	GTCCTCCGTGATGAGATTACTCTG		
CRU1	Forward	GTAGGTGTTGGTGGTGGTTTC	174	AF430071
	Reverse	CTCGCAGCAGTAGGCTTGAC		
LYZ1	Forward	TACGCGACCGATTACTGGCTAC	138	ABD65298
	Reverse	AGTCTTTGCTGCGACCACATTC		
PEN4	Forward	GGTGCGATGTATGCTACGGAA	106	DQ206402
	Reverse	CATCGTCTTCTCCATCAACCA		

## Data Availability

The data supporting the findings of this study are available from the corresponding author upon reasonable request.

## References

[1] Jennings S., Stentiford G.D., Leocadio A.M., Jeffery K.R., Metcalfe J.D., Katsiadaki I., Neil A.A., Stephen C.M., John K.P., Tim E. (2016). Aquatic food security: Insights into challenges and solutions from an analysis of interactions between fisheries, aquaculture, food safety, human health, fish and human welfare, economy and environment. Fish Fish..

[2] Fajardo C., Martinez-Rodriguez G., Costas B., Mancera J.M., Fernandez-Boo S., Rodulfo H., Donato M.D. (2022). Shrimp immune response: A transcriptomic perspective. Rev. Aquacult..

[3] JH P., Ys L., Lee S., Lee Y. (1998). An infectious viral disease of penaeid shrimp newly found in Korea. Dis. Aquat. Organ..

[4] Stentiford G.D., Lightner D.V. (2011). Cases of White Spot Disease (WSD) in European shrimp farms. Aquaculture.

[5] Flegel T.W. (1997). Major viral diseases of the black tiger prawn (*Penaeus monodon*) in Thailand. World J. Microb. Biot..

[6] CF L., Ho C.H., Peng S.E., Chen C.H., Hsu H.C., Chiu Y.L., Chang C.F., Liu K.F., Su M.S., Wang C.H. (1996). White spot syndrome baculovirus (WSBV) detected in cultured and captured shrimp, crabs and other arthropods. Dis. Aquat. Organ..

[7] Lightner D.V., Redman R.M., Pantoja C.R., Tang K., Noble B.L., Schofield P., Mohney L.L., Nunan L.M., Navarro S.A. (2012). Historic emergence, impact and current status of shrimp pathogens in the Americas. J. Invertebr. Pathol..

[8] Liu L., Shan L.P., Yan M.C., Liu G.L., Chen J. (2021). Infection of WSSV shows potential promise of a novel antiviral amino nitrophenyl medicine for application in culture of shrimp seedling. Aquaculture.

[9] Park J.H., Seok S.H., Cho S.A., Baek M.W., Lee H.Y., Kim D.J., Kim H.Y., Chang S.O., Park J.H. (2004). Safety and protective effect of a disinfectant (STEL water) for white spot syndrome viral infection in shrimp. Dis. Aqua. Organ..

[10] Lulijwa R., Rupia E.J., Alfaro A.C. (2020). Antibiotic use in aquaculture, policies and regulation, health and environmental risks: A review of the top 15 major producers. Rev. Aqua..

[11] Ring E., Olsen R.E., Gifstad T.Ø., Dalmo R.A., Amlund H., Hemre G.I., Bakke A.M. (2010). Prebiotics in aquaculture: A review. Aquacult. Nutr..

[12] Reverter M., Bontemps N., Lecchini D., Banaigs B., Sasal P. (2014). Use of plant extracts in fish aquaculture as an alternative to chemotherapy: Current status and future perspectives. Aquaculture.

[13] Stratev D., Zhelyazkov G., Noundou X.S., Krause R.W. (2018). Beneficial effects of medicinal plants in fish diseases. Aquacult. Int..

[14] Afe O.E., Omosowone O.O. (2019). Growth and feed utilization in *Clarias gariepinus* fingerlings fed on *Acacia auriculiformis* leaf supplemented diets. Int. J. Fish. Aquac..

[15] Beltrán J.M.G., Espinosa C., Guardiola F.A., Esteban M.Á. (2018). In vitro effects of *Origanum vulgare* leaf extracts on gilthead seabream (*Sparus aurata* L.) leucocytes, cytotoxic, bactericidal and antioxidant activities. Fish Shellfish Immunol..

[16] Palanikumar P., Daffni B.D.J., Lelin C., Thirumalaikumar E., Michaelbabu M., Citarasu T. (2018). Effect of *Argemone mexicana* active principles on inhibiting viral multiplication and stimulating immune system in Pacific white leg shrimp *Litopenaeus vannamei* against white spot syndrome virus. Fish Shellfish Immunol..

[17] Citarasu T., Sivaram V., Immanuel G., Rout N., Murugan V. (2006). Influence of selected Indian immunostimulant herbs against white spot syndrome virus (WSSV) infection in black tiger shrimp, *Penaeus monodon* with reference to haematological, biochemical and immunological changes. Fish Shellfish Immunol..

[18] Sanchez-Paz A. (2010). White spot syndrome virus: An overview on an emergent concern. Vet. Res..

[19] Chen W.C., Liu L., Shen Y.F., Hu Y., Ling F., Wang G.X., Zhu B. (2018). A new coumarin derivative plays a role in rhabdoviral clearance by interfering glycoprotein function during the early stage of viral infection. Cell Signal..

[20] Hu Y., Liu L., Li B., Shen Y., Wang G.X., Zhu B. (2019). Synthesis of arctigenin derivatives against infectious hematopoietic necrosis virus. Eur. J. Med. Chem..

[21] Yu Q., Liu M., Xiao H., Wu S., Qin X., Lu Z., Shi D., Li S., Mi H., Wang Y. (2019). The inhibitory activities and antiviral mechanism of *Viola philippica* aqueous extracts against grouper iridovirus infection in vitro and in vivo. J. Fish. Dis..

[22] Huang A.G., Tan X.P., Cui H.B., Qi X.Z., Zhu B., Wang G.X. (2020). Antiviral activity of geniposidic acid against white spot syndrome virus replication in red swamp crayfish *Procambarus clarkii*. Aquaculture.

[23] Bachere E., Gueguen Y., Gonzalez M., Lorgeril J., Garnier J., Romestand B. (2004). Insights into the anti-microbial defense of marine invertebrates: The penaeid shrimps and the oyster *Crassostrea gigas*. Immunol. Rev..

[24] Rajkumar T., Taju G., Majeed S.A., Sajid M.S., Kumar S.S., Sivakumar S., Hameed A.S. (2017). Ontogenetic changes in the expression of immune related genes in response to immunostimulants and resistance against white spot syndrome virus in *Litopenaeus vannamei*. Dev. Comp. Immunol..

[25] Cantelli L., Goncalves P., Guertler C., Kayser M., Pilotto M.R., Barracco M.A., Perazzolo L.M. (2019). Dietary supplementation with sulfated polysaccharides from *Gracilaria birdiae* promotes a delayed immunostimulation in marine shrimp challenged by the white spot syndrome virus. Aquacult. Int..

[26] Shan L.P., Hu L.H., Zhao Q., Yan M.C., Chen J.P. (2021). Difference in medication pattern potentially enhances antiviral efficiency of a novel amino-fluorophenyl compound on WSS risk in shrimp seedling culture. Aquaculture.

[27] Dhanasekaran S., Doherty T.M., Kenneth J. (2010). Comparison of different standards for real-time PCR-based absolute quantification. J. Immunol. Methods.

[28] Liu L., Song D.W., Liu G.L., Shan L.P., Qiu T.X., Chen J. (2020). Hydroxycoumarin efficiently inhibits spring viraemia of carp virus infection *in vitro* and *in vivo*. Zool. Res..

[29] Wang H., Qiu T.X., Lu J.F., Liu H.W., Hu L., Liu L., Chen J. (2021). Potential aquatic environmental risks of trifloxystrobin: Enhancement of virus susceptibility in zebrafish through initiation of autophagy. Zool. Res..

[30] Livak K.J., Schmittgen T.D. (2001). Analysis of relative gene expression data using real-time quantitative PCR and the 2(-Delta Delta C(T)) Method. Methods.

[31] Qiu W., Geng R., Zuo H.L., Wang S.P., He J.G., Xu X.P. (2021). Toll receptor 2 (Toll2) positively regulates antibacterial immunity but promotes white spot syndrome virus (WSSV) infection in shrimp. Dev. Comp. Immunol..

[32] Yuan F.H., Chen Y.G., Zhang Z.Z., Yue H.T., Bi H.T., Yuan K., Weng S.P. (2016). Down-regulation apoptosis signal-regulating kinase 1 gene reduced the *Litopenaeus vannamei* hemocyte apoptosis in WSSV infection. Fish Shellfish Immunol..

[33] Li H., Yin B., Wang S., Fu Q.H., Xiao B., Lu K., He J.G., Li C.Z. (2018). RNAi screening identifies a new Toll from shrimp *Litopenaeus vannamei* that restricts WSSV infection through activating Dorsal to induce antimicrobial peptides. PLoS Pathog..

[34] Pirarat N., Pinpimai K., Endo M., Katagiri T., Ponpornpisit A., Chansue N., Maita M. (2011). Modulation of intestinal morphology and immunity in Nile tilapia (*Oreochromis niloticus*) by *Lactobacillus rhamnosus* GG. Res. Vet. Sci..

[35] Luo L., Chen X.X., Cai X.F. (2001). Effects of *Andrographis paniculate* on the variation of intestinal microflora of *Ctenopharyngodon idellus*. J. Fish. China.

[36] Verbruggen B., Bickley L.K., Aerle R., Bateman K.S., Stentiford G.D., Santos E.M., Tyler C.R. (2016). Molecular mechanisms of white spot syndrome virus infection and perspectives on treatments. Viruses.

[37] Pradeep B., Rai P., Mohan S.A., Shekhar M.S., Karunasagar I. (2012). Biology, Host Range, Pathogenesis and Diagnosis of White spot syndrome virus. Indian J. Virol..

[38] Shan L.P., Zhang X., Hu Y., Liu L., Chen J. (2022). Antiviral activity of esculin against white spot syndrome virus: A new starting point for prevention and control of white spot disease outbreaks in shrimp seedling culture. J. Fish. Dis..

[39] Shan L.P., Zhou Y., Yan M.C., Liu L., Chen J., Chen J.P. (2021). A novel antiviral coumarin derivative as a potential agent against WSSV infection in shrimp seedling culture. Virus Res..

[40] Chen K., Hsu T., Huang P., Kang S., Lo C., Huang W., Chen L. (2009). *Penaeus monodon* chitin binding protein (PmCBP) is involved in white spot syndrome virus (WSSV) infection. Fish Shellfish Immunol..

[41] Li L., Lin S., Yang F. (2006). Characterization of an envelope protein (VP110) of White spot syndrome virus. J. Gen. Virol..

[42] Liu L., Qiu T.X., Song D.W., Shan L.P., Chen J. (2020). Inhibition of a novel coumarin on an aquatic rhabdovirus by targeting the early stage of viral infection demonstrates potential application in aquaculture. Antivir. Res..

[43] Tjwa E.T.T.L., Zoutendijk R., VanOord G.W., Boeijen L.L., Reijnders J.G.P., Van-Campenhout M.J.H., Knegt R.J., Janssen H.L.A., Woltman A.M., Boonstra A. (2016). Similar frequencies, phenotype and activation status of intrahepatic NK cells in chronic HBV patients after long-term treatment with tenofovir disoproxil fumarate (TDF). Antivir. Res..

[44] Citarasu T. (2010). Herbal biomedicines: A new opportunity for aquaculture industry. Aquac. Int..

[45] Zhang Y., Bian Y., Cui Q., Yuan C., Lin Y., Li J., Meng P. (2021). Effect of dietary complex Chinese herbal medicine on growth performance, digestive enzyme activities in tissues and expression of genes involved in the digestive enzymes and antioxidant enzymes and bacterial challenge in *Litopenaeus vannamei*. Aquac. Res..

[46] Wang M., Gang P., Qin Q., Tang S. (2008). Effects of chinese herbal medicine on the immunity function of *Macrobrachium nipponense* as additive in feed. J. Aqua..

[47] Liu Y., Tong B., Wang S., Li G., Tan Y., Yu H., Yu Y. (2021). A mini review of Yu-Ping-Feng polysaccharides: Their biological activities and potential applications in aquaculture. Aquac. Rep..

[48] Huang A.G., Tu X., Qi X.Z., Ling F., Zhu B., Wang G.X. (2019). *Gardenia jasminoides* ellis inhibit white spot syndrome virus replication in red swamp crayfish *Procambarus clarkii*. Aquaculture.

[49] Jiravanichpaisal P., Lee B.L., Söderhäll K. (2006). Cell-mediated immunity in arthropods: Hematopoiesis, coagulation, melanization and opsonization. Immunobiology.

[50] Kulkarni A., Krishnan S., Anand D., Kokkattunivarthil U.S., Otta S.K., Karunasagar I., Kooloth V.R. (2021). Immune responses and immunoprotection in crustaceans with special reference to shrimp. Rev. Aquacult..

[51] Peters C.W., Kruse U., Pollwein R., Grzeschik K.H., Sippel A.E. (1989). The human lysozyme gene: Sequence organization and chromosomal localization. Eur. J. Biochem..

[52] Kaizu A., Fagutao F.F., Kondo H., Aoki T., Hirono I. (2011). Functional analysis of C-type lysozyme in penaeid shrimp. J. Biol. Chem..

[53] Liu H., Jiravanichpaisal P., Söderhäll I., Cerenius L., Söderhäll K. (2006). Antilipopolysaccharide factor interferes with white spot syndrome virus replication in vitro and in vivo in the crayfish *Pacifastacus leniusculus*. J. Virol..

[54] Suraprasit S., Methatham T., Jaree P., Phiwsaiya K., Senapin S., Hirono I., Lo C.F., Tassanakajon A., Somboonwiwat K. (2014). Anti-lipopolysaccharide factor isoform 3 from *Penaeus monodon* (ALFPm3) exhibits antiviral activity by interacting with WSSV structural proteins. Antivir. Res..

[55] Khairnar K., Raut M.P., Chandekar R.H., Sanmukh S.G., Paunikar W.N. (2013). Novel bacteriophage therapy for controlling metallo-beta-lactamase producing *Pseudomonas aeruginosa* infection in catfish. BMC Vet. Res..

[56] Lomelí-Ortega C.O., Martínez-Díaz S.F. (2014). Phage therapy against *Vibrio parahaemolyticus* infection in the whiteleg shrimp (*Litopenaeus vannamei*) larvae. Aquaculture.

[57] Hameed A.S., Balasubramanian G., Musthaq S.S., Yoganandhan K. (2003). Experimental infection of twenty species of Indian marine crabs with white spot syndrome virus (WSSV). Dis. Aquat. Organ..

[58] Dey B.K., Dugassa G.H., Hinzano S.M., Bossier P. (2020). Causative agent, diagnosis and management of white spot disease in shrimp: A review. Rev. Aquacult..

